# Genetic Diversity and Variability in Endangered Pantesco and Two Other Sicilian Donkey Breeds Assessed by Microsatellite Markers

**DOI:** 10.1100/2012/648427

**Published:** 2012-04-29

**Authors:** Salvatore Bordonaro, Anna Maria Guastella, Andrea Criscione, Antonio Zuccaro, Donata Marletta

**Affiliations:** DISPA, Sezione di Scienze delle Produzioni Animali, Università degli studi di Catania, Via Valdisavoia 5, 95123 Catania, Italy

## Abstract

The genetic variability of Pantesco and other two Sicilian autochthonous donkey breeds (Ragusano and Grigio Siciliano) was assessed using a set of 14 microsatellites. The main goals were to describe the current differentiation among the breeds and to provide genetic information useful to safeguard the Pantesco breed as well as to manage Ragusano and Grigio Siciliano. In the whole sample, that included 108 donkeys representative of the three populations, a total of 85 alleles were detected. The mean number of alleles was lower in Pantesco (3.7), than in Grigio Siciliano and Ragusano (4.4 and 5.9, resp.). The three breeds showed a quite low level of gene diversity (He) ranging from 0.471 in Pantesco to 0.589 in Grigio. The overall genetic differentiation index (Fst) was quite high; more than 10% of the diversity was found among breeds. Reynolds' (*D*_*R*_) genetic distances, correspondence, and population structure analysis reproduced the same picture, revealing that, (a) Pantesco breed is the most differentiated in the context of the Sicilian indigenous breeds, (b) within Ragusano breed, two well-defined subgroups were observed. This information is worth of further investigation in order to provide suitable data for conservation strategies.

## 1. Introduction

The donkey (*Equus asinus*) was domesticated about 6000 years ago starting from one or two subspecies of African wild asses (*E. africanus*) [1, 2]. For many centuries donkey has been used as beast of burden in many cultures. Today donkeys and mules are still essential for transportation of heavy load, people, and possessions in poor, arid, and rough regions of the world [3], but in the developed country, this pack animal is no longer required. As a consequence, not only individual breeds are endangered, but also the whole species is heading for extinction [4].

In Italy, six donkey breeds are already extinct; in contrast during the last few years, due to the exploitation of donkey's products (milk and meat) many local breeds and populations are growing in census. Moreover some small populations are undergoing conservation programmes.

Pantesco breed represents an emblematic case: this ancient breed was imported by the Arabs to the isle of Pantelleria [5] in the Sicilian channel. For long time it was employed in agriculture on the rugged paths of the island. Pantesco has a short haired and fine brown coat, with white belly, muzzle, and eye rings. The withers height is about 125–130 cm; the type is dolichomorphic. This donkey, able to move in the fast and sure “Tölt-gait”, is well adapted to harsh environment [6].

The employment of Pantesco stallions in Sicilian stud farms is documented since 1926. This breed was also used in the breeding of mules, employed in the army, and exported to USA and Greece.

After the Second World War, because of the mechanization in agriculture, the exportation, and the establishment of the stud book of Ragusano, Pantesco became severely threatened with extinction.

In the last twenty years the Sicilian Forest Administration (Azienda Foreste Demaniali di Trapani) carried out a morphologic and genetic selection on more than 200 potential Pantesco crossbreed reared in Egadi Island and Trapani Province. Nine donkeys (three males and six females) were identified and used as founders to reconstitute the breed. Embryo transfer (ET) was also employed as a tool in the conservation project [7].

Today about 47 recorded Pantesco donkeys are bred in the San Matteo Farm of Erice (Trapani). Within the frame of breed conservation, genetic characterization is important with regard to breed integrity and represents an essential prerequisite for handling genetic resources [8]. Microsatellite markers proved to be a reliable and frequently used tool to quantify genetic variation within and among breeds and useful for the conservation management of animal populations [9].

A preliminary characterization of the Sicilian donkeys, mainly focused on the genetic analysis of Pantesco, was performed using a set of 11 microsatellites [10] and genealogical data [11].

The aim of this study was to measure the genetic diversity and variability in the three Sicilian indigenous donkey breeds (Pantesco, Ragusano, and Grigio Siciliano), using molecular information supplied by a set of 14 microsatellite markers, in order to provide suitable data for breeding schemes and conservation strategies.

## 2. Material and Methods

### 2.1. Sampling and DNA Extraction

Blood (10 mL) was sampled in K3-EDTA tubes from 108 Sicilian donkeys (39 Pantesco, 53 Ragusano, 16 Grigio Siciliano) reared all over Sicily. Sampling was achieved among minimally related donkeys by using pedigree information when available and avoiding first- and second-order relatives. In the case of Pantesco breed, the sample consisted of nearly the entire Stud Book-registered population (47 heads) reared in the “San Matteo Farm” in Erice (Trapani). Forty six horses belonging to Sicilian Oriental Purebred (*E. caballus*) were added to the data set and used as out-group in phylogenetic analysis.

DNA was extracted using “Illustra blood genomic Prep Mini Spin” kit (GE Healthcare, Little Chalfont, UK) and then checked for quality and concentration by NanoDrop ND 1000 spectrophotometer (Thermo Fisher Scientific, Wilmington, USA).

### 2.2. Microsatellite Amplification and Analysis

The whole sample (108 donkeys and 46 horses) was genotyped through a set of 14 microsatellite markers (AHT4, AHT5, ASB23, HMS2, HMS3, HMS5, HMS6, HMS7, HTG4, HTG6, HTG7, HTG10, HTG15, VHL20) amplified in three PCR multiplex reactions, using a PE GeneAmp PCR 9600 system thermocycler (Applied Biosystems, Foster City, CA, USA). Fluorescent-labelled PCR products were diluted, mixed with an internal size standard, and analysed by the automatic AB3130 DNA Sequencer equipped with GeneScan and Genotyper software (Applied Biosystems, Foster City, CA, USA). Microsatellite markers were chosen, on the base of their degree of information obtained on a smaller sample, among those reported in the literature leading with donkey [12] and horse [13] biodiversity.

### 2.3. Statistical Analysis

Individual multilocus genotypes were processed by means of GENALEX v.6.4 platform [14] to perform file conversions and calculate the main parameters of genetic variability. For each *locus* and breed and on the whole sample, the allele frequencies, private alleles (*A*_*p*_), and observed (*H*_*o*_) and unbiased expected (*H*_*e*_) heterozygosities were calculated.

The polymorphism information content (PIC) for each *locus* and breed was calculated [15].

Hardy-Weinberg equilibrium was tested by the software Genepop v.4.0 [16] which was used to perform the score test per *locus *and breed and global tests across *loci* and across sample; tests were implemented using the Markov chain algorithm (10000 dememorizations, 5000 batches, and 5000 iterations per batch).

The presence of null alleles was tested with MICRO-CHECKER v.2.2.3 [17], using the methods by Chakraborty et al. [18] and Brookfield [19].

FSTAT v.2.9.3 software [20] was used to estimate the *F*-statistics [21] and their significance as well as the rarefacted number of alleles (Ar) based on the minimum sample size.

The significance levels obtained from multiple tests, carried out for HW-Equilibrium and *F*-statistics, were corrected by the sequential Bonferroni method [22] to reduce the occurrence of type I error.

In order to measure the short-term divergence of the donkey breeds, the Reynolds' (*D*_*R*_) pairwise genetic distances [23] were calculated by PHYLIP ver.3.69 package [24]. Moreover, the Neighbour-Joining algorithm was implemented on *D*_*R*_ and the strength of the nodes was based on 1000 bootstrap resamplings of the allelic frequencies.

The model-based approach proposed in the software STRUCTURE 2.3 [25] was used to assess the genomic clustering of the sample. As suggested by the authors for populations with possible mixed ancestry, the admixture model associated to the option of correlated allele frequencies [26] was implemented to infer the populations' structure using no prior information. Running length was set to 500000 burn-ins followed by 500000 iterations. The range of possible clusters (*K*) tested was from 1 to 10 and 10 different runs were carried out for each *K*. The number of clusters fitting best our data was established by plotting the mean ln⁡ Pr(*X* | *K*) over the multiple independent runs for each *K*, as suggested by the authors.

The correspondence analysis in which the Chi-square distances measure the proximity of the taxa was performed by GENETIX v.4.05 software [27] and breeds and individuals were spatially plot in accordance with allele frequencies.

## 3. Results

The 14 microsatellite markers resulted to be polymorphic in the whole sample and in each breed but for HMS5 in Pantesco (Table 1). A total of 85 alleles were observed in the three Sicilian donkey breeds, with the number of alleles ranging from 2 to 11 (6.07 on average). The observed heterozygosity (*H*_*o*_) varied between 0.154 (HMS5) and 0.736 (HTG10), while the expected heterozygosity (*H*_*e*_) ranged between 0.159 (HMS5) and 0.822 (AHT5).

The polymorphism information content (PIC) per *locus* showed only two markers with values under the 20% and an average of 0.537 (Table 1).

The significant overall *loci F*_ST_ index revealed that 10.8% of the total genetic variation observed in the sample is explained by population differences, whereas the remaining is due to the differences within subpopulations. The *locus* which contributed most to the differentiation of the samples was HMS3, while HTG4 resulted to be a nondiscriminating marker (Table 2).

The highly significant (*P* < 0.001) Fis value (0.161) revealed a rather high inbreeding degree within breeds. In particular, six loci gave a relevant significant contribution to the total inbreeding index (Fit = 0.251), with a high heterozygote deficit within breeds.

Mean number of alleles (MNA), allelic richness, Fis, and heterozygosities per breed are reported in Table 3. Ragusano breed showed the highest number of alleles (82), Pantesco the lowest (52). Pantesco highlighted the lowest genetic variability for all the parameters inferred per breed.

A total of 18 breed-specific alleles were observed: 15 in Ragusano, 2 in Pantesco, and 1 in Grigio Siciliano, always at a frequency lower than 10% (data not shown).

Fis values, calculated per breed, indicated a moderate deficit of heterozygosity in all the three genetic types, probably due to a departure from random mating.

In the whole sample a significant deviation from HW-equilibrium was observed (*P* < 0.001). At breed level an excess of homozygotes was detected at 3 *loci* in Pantesco, 3 in Ragusano, and 1 in Grigio Siciliano. Only one out of 14 microsatellite markers (HMS2) was not consistently in Hardy-Weinberg equilibrium in all the three breeds, so that it was excluded from the clustering analysis.

**Figure 1:**
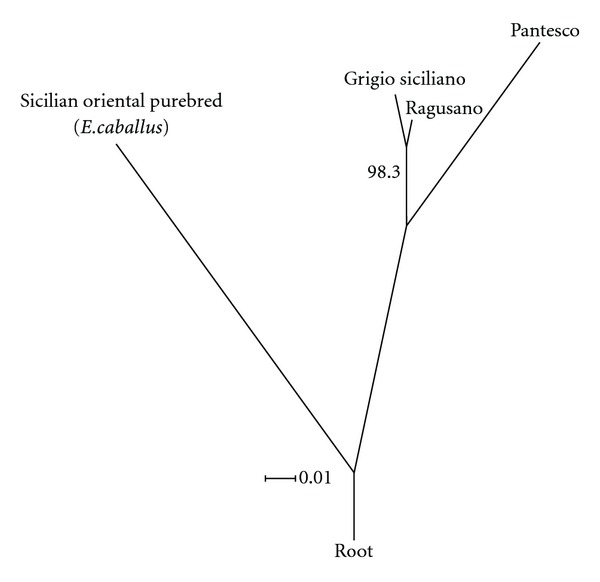
Neighbour-Joining rooted tree built on *D*_*R*_ genetic distances. This is a consensus tree out of the trees resulted from 1000 bootstrap resamplings of the allele frequencies at 13 *loci*'s.

The topology of the Neighbour Joining tree (Figure 1), built on *D*_*R*_ genetic distances, clearly highlighted the genetic differentiation of Pantesco breed (average *D*_*R*_ = 0.104) in comparison with Ragusano and Grigio Siciliano which were the closest breeds (*D*_*R*_ = 0.032) and significantly clustered (98.3% node support).

**Figure 2:**
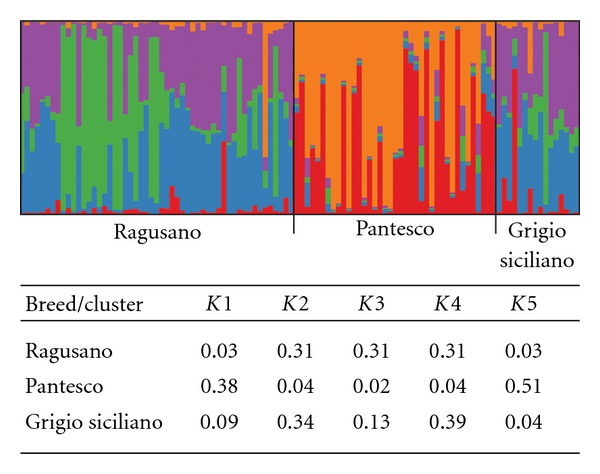
Breeds' genome distribution into the *K* = 5 inferred clusters. Analysis, conducted on the allele frequencies of 13 *loci*.

The clustering analysis using the Bayesian model approach was conducted on 13 microsatellite markers and under the hypothesis that donkey breeds had an ancestral common origin. The mean estimated ln⁡ probability of data (ln⁡ Pr(*X* | *K*)) for each inferred cluster was plotted (data not shown) and suggested *K* = 5 as the number of ancestral clusters that captures most of the structure in the sample. Breeds' genome fractions versus the five inferred clusters are reported in Figure 2. For *K* = 5, Pantesco's genome is mainly distributed into two clusters (1 and 5) with a total membership close to 90%, Ragusano's resulted to be equally shared into the clusters 2, 3, and 4 (total 93.5%), while Grigio Siciliano breed mostly belonged to the clusters 2 and 4 (73.84%).

The results of the admixture analysis, reported for *K* = 5, highlighted the clear differentiation of Pantesco breed from the other two Sicilian breeds and at the same time a substructuring within the Ragusano in comparison to the rest of the sample. These outcomes were apparent along the analysis range from *K* = 3 to *K* = 10, while in correspondence with *K* = 2 only the originality of Pantesco was visible.

**Figure 3:**
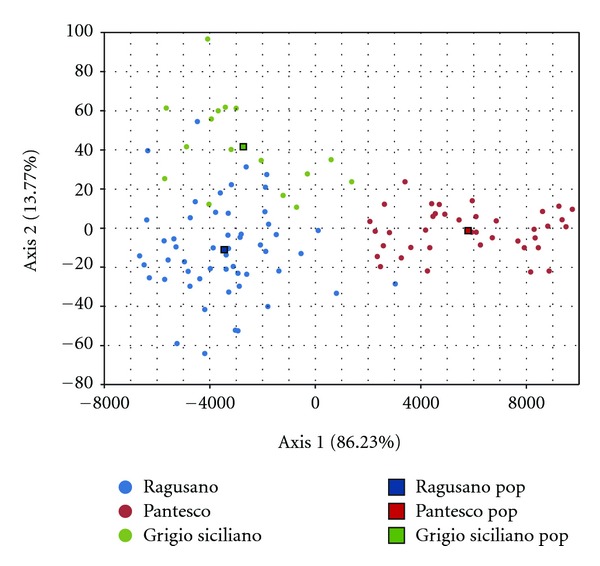
Plot of the correspondence analysis on allele frequencies of 13 *loci* of the three Sicilian donkey breeds.

Correspondence analysis provided an alternative spatial representation of breeds and individuals scattered in the metric space (Figure 3). The first axis, which contributed mostly (86.23%) to the total inertia, led the Pantesco donkeys to form a well-defined group, while Ragusano and Grigio Siciliano showed their close relationship.

## 4. Discussion

Genetic characterization studies dealing on donkey species are scant and focused mainly on Mediterranean and Asian breeds. In terms of mean number of alleles, the genetic variability observed in the three Sicilian donkey breeds was lower than that reported in five Spanish breeds [12] and three Croatian breeds [28], but higher than that observed in the Amiata donkey from Italy [29]. In our sample, expected heterozygosity was lower than that inferred in European breeds in the above-mentioned studies [12–29] and in eight Chinese breeds [30]. This outcome is reasonable if we consider the presence of Pantesco: this breed is undergoing genetic recovery, starting from a small nucleus of 9 founders but the actual total number of heads makes it as an endangered breed with a low genetic variability. Notwithstanding this, the overall Fst index showed a good rate of differentiation at population level: the value of 10.8% was higher than that reported for Catalana breed [31] and five Spanish breeds [23].

Molecular characterization of Sicilian breeds revealed a high degree of internal structuring, highlighted by the high and significant Fst indexes per *locus*. This evidence can be mainly imputable to Pantesco's structure which clearly differentiated from Ragusano's and Grigio Siciliano's. The marked differentiation of Pantesco seems to be a sign of the appropriate plan of genetic management carried out so far.

The observed *loci* polymorphism, despite the low PIC values, made the breeds' differentiation possible. The pairwise genetic distances and the Neighbour-Joining tree highlighted the close genetic relationship between Ragusano and Grigio Siciliano, which defined a distinct cluster from Pantesco. This result is supported by the historical records which reports that until 1950 stallions reared in Sicily had both bay and grey coat color (the coat colors of the current Ragusano and Grigio Siciliano, resp.) and the morphological differentiation was based only on body size with respect to the breeding area [32]. Only starting from 1953, when Ragusano's Stud-book was established, Sicilian donkeys characterized by the grey coat color were excluded from selection and they were consigned to a marginal role.

The admixture analysis led to the identification of an interesting and useful result regarding the genomic structure of the analyzed sample. With regard to the presented results (*K* = 5), anyhow consistent along the analyzed *K* range, Ragusano breed presented about 30% of its genome's fraction which belong to a exclusive cluster: this might represent the most selected nucleus of the breed; at the same time, the remaining genome's structure is in common with that of Grigio Siciliano, confirming the occurred gene flow between them and their common origin. Structure analysis clearly shows that Pantesco's genome is grouped in exclusive clusters for almost the 90%. This data strengthens the originality of this breed in the context of the Sicilian indigenous donkey breeds.

Sicilian donkey breeds and populations are already classified as endangered. The low genetic variability, observed in Ragusano, Grigio Siciliano, and particularly expected in Pantesco, makes further safeguard and management plans compelling. Exploitation management should be realized by increasing the number of official stallions and reduce as low as possible the inbreeding rate at mating, particularly in those farms specialized in milk production which account for more than 50 females.

In the case of Pantesco breed, the possibility of admitting in its selection schemes reproducing females from Ragusano breed appears advantageous in order to widen the current genetic pool.

Safeguard protocols need to be accomplished before the inbreeding rate brings a marked fitness reduction and leads to an increasing frequency of genetic diseases, reproductive disorders, and a general drop of vital and productive performance [33].

## Figures and Tables

**Table 1:** Number of alleles, observed (*H*_*o*_) and expected heterozygosity (*H*_*e*_), and PIC values per *locus* inferred on the whole sample of three Sicilian donkey breeds.

Locus	*N*° of alleles	*H*_*o*_	*H*_*e*_	PIC
AHT4	8	0.533	0.747	0.703
AHT5	10	0.581	0.822	0.792
ASB23	5	0.704	0.740	0.692
HMS2	8	0.271	0.582	0.533
HMS3	8	0.463	0.611	0.558
HMS5	2	0.154	0.159	0.146
HMS6	4	0.250	0.483	0.393
HMS7	6	0.222	0.310	0.291
HTG10	7	0.736	0.794	0.760
HTG15	3	0.514	0.553	0.482
HTG4	3	0.178	0.194	0.176
HTG6	5	0.556	0.674	0.608
HTG7	11	0.648	0.817	0.796
VHL20	5	0.561	0.652	0.591
Average	6.07	0.455	0.581	0.537

SE	±0.722	±0.054	±0.059	±0.06

**Table 2:** *F*-statistics (Fit, Fst, and Fis) per *locus* and overall the three Sicilian donkey breeds.

Locus	Fit	Fst	Fis
AHT4	0.322**	0.123**	0.227**
AHT5	0.342**	0.153**	0.223**
ASB23	0.082	0.091**	−0.01
HMS2	0.547**	0.067**	0.515**
HMS3	0.315**	0.244**	0.093
HMS5	0.056	0.065*	−0.009
HMS6	0.496**	0.064	0.461**
HMS7	0.292**	0.032	0.268*
HTG10	0.093	0.053**	0.042
HTG15	0.096	0.071**	0.027
HTG4	0.081	−0.009	0.089
HTG6	0.234**	0.18**	0.066
HTG7	0.229**	0.071**	0.171**
VHL20	0.182*	0.124**	0.066

All	0.251^†††^	0.108^†††^	0.161^†††^

Adjusted nominal levels (Bonferroni): *P < 0.05/14; **P < 0.01/14; ***P < 0.001/14; ^†††^ *P* < 0.001.

**Table 3:** Number of individuals, mean number of alleles (MNAs), allelic richness (Ar), observed (*H*_*o*_) and expected (*H*_*e*_) heterozygosities, and Fis inferred per breed in three Sicilian donkey sample.

Breed	*N*	MNA	Ar	*H*_*o*_	*H*_*e*_	Fis
Ragusano	53	5.857	3.8	0.496	0.579	0.144***
SE		±0.69		±0.058	±0.057	
Pantesco	39	3.714	3.0	0.385	0.471	0.185***
SE		±0.47		±0.073	±0.066	
Grigio Siciliano	16	4.429	3.7	0.496	0.589	0.162***
SE		±0.48		±0.057	±0.057	

Adjusted nominal levels (Bonferroni): ***P < 0.001/3.
